# Fever and hypothermia represent two populations of sepsis patients and are associated with outside temperature

**DOI:** 10.1186/s13054-021-03776-2

**Published:** 2021-10-21

**Authors:** Daniel O. Thomas-Rüddel, Peter Hoffmann, Daniel Schwarzkopf, Christian Scheer, Friedhelm Bach, Marcus Komann, Herwig Gerlach, Manfred Weiss, Matthias Lindner, Hendrik Rüddel, Philipp Simon, Sven-Olaf Kuhn, Reinhard Wetzker, Michael Bauer, Konrad Reinhart, Frank Bloos, the MEDUSA study group, Gernot Marx, Achim Schindler, Tobias Schürholz, Heike Schlegel-Höfner, Gunther Lehmann, Annett Sander, Steffen Friese, Christian Scholz, Pia Fischer, Christina Fuchs, Lutz Becher, Norbert Salewsky, Torsten Schreiber, Anton Goldmann, Didier Keh, Katrin Schmid, Winfried Menning, Renate Steuckart, Robert Barz, Karin Dey, Meike Fahrenholz, Martin Müller, Herwig Gerlach, Susanne Toussaint, Jörg Brederlau, Friedhelm Bach, Dirk Buschmann, Ingo Gummelt, J. Hoeschen, Marion Klaproth, Ina Vedder, Ulrike Bachmann-Holdau, Jürgen Eiche, Rolf Hauschild, Martina Lange, Davia Herrmann-Karbaum, Annette Lubasch, Marcus Rücker, Christian Icke, Alexander Lucht, Andreas Meier-Hellmann, Jan Wagner, Olaf Arnold, Steffen Kästner, Tobias Clausen, Michael Sternkopf, Robert Voswinckel, T. Benndorf, Christel Eiserloh, Gerhard Kuhnle, Mathias Koch, Manuela Gerber, Matthias Gründling, Liane Guderian, Sven-Olaf Kuhn, Christian Scheer, Gerd Scheiber, Frank Bloos, Susann Christink, Martina Kortegast, Claudia Matthäus-Krämer, Marcel Mücke, Bernhard Poidinger, Hendrik Rüddel, Ulrike Redlich, Daniel Schwarzkopf, Daniel Thomas-Rüddel, Christel Volkmer, Stefanie D’Aria, Thees Lemke, Birgit Michaelsen, Dirk Schädler, Nina Schulz-Ruhtenberg, Norbert Weiler, Martin Anetseder, Zoran Textor, Udo Kaisers, Philipp Simon, Georg Braun, Nicole Jensen, Werner Gegenfurtner, Alexander Meinhardt, Robert Schmitt, Andrea Teichert, Klaus-Dieter Becker, Anja Diers, Florian Jelschen, Andreas Weyland, Frieder Knebel, Thomas Kupfer, Rüdinger Sinz, Petra Bautz, Annemarie Fischer, Armin Seibel, Christoph Fleischhacker, Helene Häberle, Philipp Henn, Friederike Mezger, Peter Rosenberger, Reimer Riessen, Silvia Ziegler, Eberhard Barth, Hendrik Bracht, I. Heymann, A. Hinder, R. Sens, Manfred Weiss, Christof Lascho, Henriette Micke, Falk Schmidt, Stefanie Schilling, Gabriele Wöbker

**Affiliations:** 1 Center for Sepsis Control and Care, Jena University Hospital, Jena, Germany; 2 Department of Anesthesiology and Intensive Care Medicine, Jena University Hospital, Am Klinikum 1, 07747 Jena, Germany; 3 Potsdam Institute for Climate Impact Research, Potsdam, Germany; 4 Department of Anesthesiology and Intensive Care Medicine, Greifswald University Hospital, Greifswald, Germany; 5 Department of Anesthesiology and Intensive Care Medicine, Evangelisches Klinikum Bethel, Bielefeld, Germany; 6 Department of Anesthesiology and Intensive Care Medicine, Vivantes Klinikum Neuköln, Berlin, Germany; 7 Department of Anesthesiology and Intensive Care Medicine, Ulm University Hospital, Ulm, Germany; 8 Department of Anesthesiology and Intensive Care Medicine, University Hospital Schleswig-Holstein, Kiel, Germany; 9 Department of Anesthesiology and Intensive Care Medicine, Leipzig University Hospital, Leipzig, Germany; 10 Department of Anesthesiology and Operative Intensive Care Medicine (CCM, CVK), Charité University Medical Center Berlin, Berlin, Germany

**Keywords:** Fever, Sepsis, Outcome, Mortality, Body temperature, PCT, Lactate, Blood cultures, Prognosis

## Abstract

**Background:**

Fever and hypothermia have been observed in septic patients. Their influence on prognosis is subject to ongoing debates.

**Methods:**

We did a secondary analysis of a large clinical dataset from a quality improvement trial. A binary logistic regression model was calculated to assess the association of the thermal response with outcome and a multinomial regression model to assess factors associated with fever or hypothermia.

**Results:**

With 6542 analyzable cases we observed a bimodal temperature response characterized by fever or hypothermia, normothermia was rare. Hypothermia and high fever were both associated with higher lactate values. Hypothermia was associated with higher mortality, but this association was reduced after adjustment for other risk factors. Age, community-acquired sepsis, lower BMI and lower outside temperatures were associated with hypothermia while bacteremia and higher procalcitonin values were associated with high fever.

**Conclusions:**

Septic patients show either a hypothermic or a fever response. Whether hypothermia is a maladaptive response, as indicated by the higher mortality in hypothermic patients, or an adaptive response in patients with limited metabolic reserves under colder environmental conditions, remains an open question.

*Trial registration* The original trial whose dataset was analyzed was registered at ClinicalTrials.gov (NCT01187134) on August 23, 2010, the first patient was included on July 1, 2011.

**Supplementary Information:**

The online version contains supplementary material available at 10.1186/s13054-021-03776-2.

## Background

The great Canadian physician Sir William Osler wrote: “*Humanity has but three great enemies: Fever, famine and war; of these by far the greatest, by far the most terrible, is fever.*” [1]. Fever is caused by release of pyrogens such as acute phase proteins and is frequently the first symptom of infection [2]. Therefore, the terms fever and infection are often used almost synonymously. But it has long been recognized that there are variable thermoregulatory responses in sepsis [3–5]. The sepsis-1 definition of the Systemic Inflammatory Response Syndrome, therefore, had included both fever and hypothermia [6]. The impact of the thermoregulatory response on prognosis is a long-lasting debate reaching back to the 1960s, but data are inconclusive. Hypothermia or fever was associated with protective or detrimental effects in animal models of severe infection or inflammation [7–10]. In a meta-analysis of clinical data, hypothermic sepsis patients showed a higher mortality than those with fever [11]. However, most included studies were small and there was a high heterogeneity.

The immunological theory of resistance versus tolerance as two distinct, well-regulated response patterns to infection gained acceptance over the last decade [12, 13]. Recent animal studies added a pathophysiological and metabolic perspective whereby hypothermia is aimed at tolerance and energy conservation and fever is aimed at pathogen clearance. Physiological fitness, feeding, environmental temperature and the degree of the immune challenge seem to be important factors promoting fever or hypothermia in animal experiments [10, 14, 15].

To evaluate whether similar processes might play a role in humans, we assessed the thermoregulatory response of septic patients and its association with predisposing factors including environmental temperature, disease severity and outcome in a secondary analysis of a large dataset [16].

## Methods

### Study design

This is a secondary analysis of the prospectively collected patient-level dataset from the MEDUSA-study—a cluster randomized quality improvement trial aiming to improve early sepsis diagnosis and treatment in the participating hospitals by a multifaceted educational program [16]. The original trial was registered at ClinicalTrials.gov (NCT01187134) and was approved by the local ethics committees (see Additional file 1 for a complete list) at each participating institution and by the responsible data protection boards.

### Study population

Patients treated between July 1, 2011, and May 31, 2015, on the participating intensive care units (ICUs) with proven or suspected infection and at least one new infection-related organ dysfunction were eligible for inclusion. Patients were excluded if they had relevant limitations of therapy, were not treated on a participating ICU or had infection control measures started at another hospital. Characteristics of participating hospitals have been described previously [16, 17]. As we performed a secondary analysis, no sample size was calculated.

### Data collection

Data collection and definitions were as previously described [16, 17]. Briefly, the onset of severe sepsis or septic shock was defined as the time of first infection-related organ dysfunction. Body temperature measurements were taken as part of routine care. A central temperature was measured rectal, tympanic or in the blood stream. For oral, axillary or groin measurements, study personnel was instructed to add 0.5 °C to approximate a central measurement. The most pathological temperature within the first 24 h after sepsis onset was recorded once for each patient. Highest values of laboratory parameters but not changes over time were recorded as baseline data within the first 24 h after the onset of severe sepsis as baseline data.

For the analysis of environmental weather conditions, we used data from the meteorological stations network of the German Meteorological Service (DWD). Daily data from 273 long-term operating weather stations since 1961 to date were selected and interpolated to a regular 12 × 12 km grid using an inverse distance weighting (IDW) with a height correction for temperature values [18], resulting in a homogeneous database that is annually expanded and also used for various applications for climate change diagnostic and monitoring on the national level [19]. Mean outside air temperature of the two days before and the day of sepsis onset were matched to cases based on postal codes of the treating hospitals, and the mean for the three days was used for all further analyses.

### Data analysis

Patients were grouped into four groups, i.e., hypothermia (≤ 35.5 °C), normothermia (35.6–37.5 °C based on published data [20]), mild fever (37.6–38.9 °C) and high fever (≥ 39 °C). Differences between these groups regarding patients’ demographic and clinical characteristics were presented by descriptive statistics and assessed by appropriate univariate tests depending on the structure and distribution of data. To investigate the relationship between body temperature and lactate, procalcitonin and mortality, as well as between body temperature and outside temperature graphical representations were used. For a more detailed description of the association to smaller temperature, groups were formed by using 1 °C intervals within the previously defined groups. As mild fever encompassed a 1.5 °C interval, it was not further divided. To better visualize the effect of extremely high fever, we used an additional cutoff at 40.5 °C. The nonlinearity of the relations was additionally investigated by using regression analyses with fractional polynomials and plotting the regression predictions [21]. One outlier in body temperature with a temperature of < 25 °C and four outliers in outside temperature with a temperature of <− 15 °C were excluded for these. To further identify predictors associated with the four body temperature groups, a multinomial logistic regression model including patient characteristics (age, sex, BMI), infection characteristics (origin and focus of infection, pathogen in blood culture), inflammatory markers (procalcitonin, WBC, leucopenia) and mean outside temperature was calculated. Predictors not significantly associated with body temperature were omitted from the final model. To achieve normal distribution for regression analysis, PCT was logarithmically transformed to the base of 10. To assess whether body temperature and mortality are independently associated, a logistic regression model predicting 28-day mortality by body temperature intervals was calculated, including patient characteristics (age, sex, BMI), infection characteristics (origin and focus of infection, pathogen in blood culture), inflammatory markers (procalcitonin, WBC, leucopenia), disease severity (SOFA score, lactate, septic shock), time to antibiotics and mean outside temperature as possibly confounding variables. Regression analyses were conducted by generalized hierarchical linear models with a random intercept to adjust for clustering of cases in hospitals. No sensitivity analyses were performed.

A p-value of less or equal to 0.05 was considered statistically significant for all tests. Estimated values are presented with 95% confidence intervals (CIs). Missing values were handled by pairwise deletion. Analyses were performed using IBM SPSS Statistics 25 (IBM, Armonk, NY), R (Version 3.4.0; R Core Team, Wien, Austria), SAS 9.4 (SAS, Cary, NC) and GraphPad Prism 9 (GraphPad Software, San Diego, CA).

## Results

### Patient characteristics

**Fig. 1:**
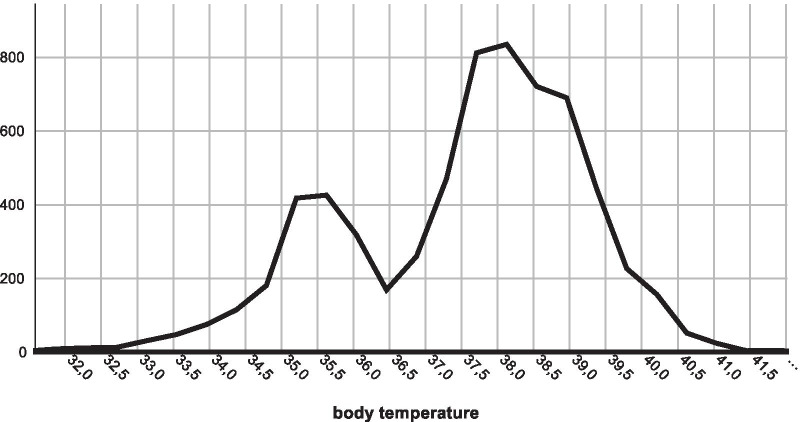
Frequency distribution of body temperature (°C) in 6542 patients with new onset of severe sepsis

**Fig. 2:**
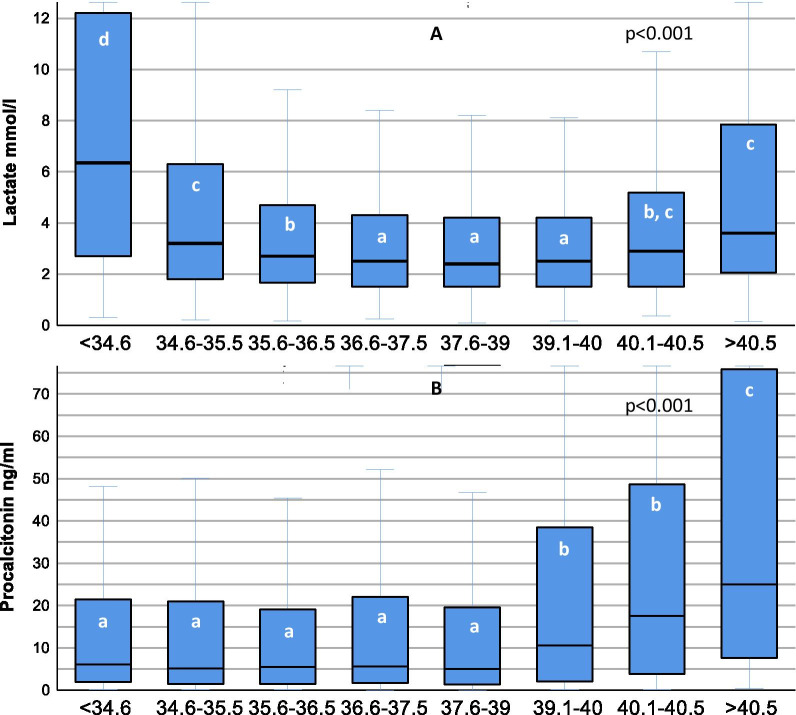
Lactate concentrations (mmol/ml, **a**) and procalcitonin concentrations (ng/ml, **b**), median and interquartile range, associated with body temperature intervals (°C); *p* value for overall difference, each white letter denotes a homogenous subgroup with increasing values from a to d, categories not sharing a common letter are significantly different from each other. Kruskal–Wallis test with stepwise post hoc comparison

During the study period, 6561 patients with severe sepsis including septic shock from 40 hospitals were documented. Body temperature was not recorded in 19 patients resulting in 6542 analyzable cases. Body temperatures showed a bimodal distribution pattern with a frequency peak at about 35.5 °C and an approximately twice times bigger peak between 38 and 38.5 °C (Fig. 1). Baseline and outcome clinical information depending on body temperature groups are shown in Table 1. Higher lactate levels were observed at both extremes of the body temperature spectrum (Fig. 2A, Additional file 2: aFigure 1), while higher procalcitonin levels were associated with increasingly higher fever (Fig. 2B) and very low body temperatures (Additional file 2: aFigure 2).

**Table 1:** Patient characteristics depending on body temperature groups

Characteristics	All patients (*n* = 6542)	Hypothermia ≤ 35.5 °C (*n* = 1042)	Normothermia 35.6–37.5 °C (*n* = 1692)	Mild fever 37.6–39 °C (*n* = 2550)	High fever ≥ 39.1 °C (*n* = 1258)	*p* value
Age (years)	70 [59–77]	72 [60–78]	77 [61–79]	70 [58–76]	67 [55–75]	< 0.001
Sex (male)	4079 (62.4)	606 (58.2)	1004 (59.3)	1635 (64.1)	834 (66.3)	< 0.001
Body mass index	26.3 [23.4–30.3]	25,5 [22.5–29.2]	26.0 [23.0–29.8]	26.6 [23.9–30.5]	27,4 [1–34]	< 0.001
Origin of infection						< 0.001
Community-acquired	2790 (42.6)	496 (47.6)	746 (44.1)	976 (38.3)	572 (45.5)	
Nosocomial (ICU)	1471 (22.5)	139 (13.3)	315 (18.6)	678 (26.6)	339 (26.9)	
Nosocomial (ward)	2112 (32.3)	367 (35.2)	593 (35.0)	835 (32.7)	317 (25.2)	
Nosocomial (nursing home)	168 (2.6)	39 (3.7)	38 (2.2)	61 (2.4)	30 (2.4)	
Focus of infection						
Respiratory	2678 (40.9)	352 (33.8)	593 (35.0)	1172 (46.0)	561 (44.6)	< 0.001
Abdominal	2422 (37.0)	473 (45.4)	756 (44.7)	896 (35.1)	297 (23.6)	< 0.001
Urogenital	869 (13.3)	125 (12.0)	221 (13.1)	288 (11.3)	235 (18.7)	< 0.001
Bones/soft tissue	851 (13.0)	156 (15.0)	249 (14.7)	306 (12.0)	140 (11.1)	0.003
Other	728 (11.1)	97 (9.3)	130 (7.7)	265 (10.4)	236 (18.8)	< 0.001
Clinical data						
Pathogen in BC	2167 (40.6)	315 (37.5)	458 (36.2)	788 (38.2)	606 (51.6)	< 0.001
PCT (ng/ml)	5.9 [1.6–23.9]	5.4 [1.6–21.1]	5.6 [1.6–20.7]	5.0 [1.3–19.5]	10.6 [2.2–39.2]	< 0.001
CRP (mg/ml)	204[115–298]	165 [94–263]	213 [119–304]	218 [125–307]	199 [108–288]	< 0.001
WBC (Gpt/l)	16.0 [10.5–22.9]	16.8 [10.9–24.5]	16.6 [11.4–23.8]	15.7 [10.3–21.9]	15.2 [9.6–22.4]	< 0.001
Leukopenia (WBC ≤ 4)	703 (10.8)	119 (11.4)	159 (9.4)	264 (10.4)	161 (12.8)	0.02
Lactate (mmol/l)	2.6 [1.6–4.8]	3.8 [1.9–8.4]	2.5 [1.6–4.5]	2.4 [1.5–4.2]	2.5 [1.5–4.4]	< 0.001
Urine output (ml/24 h)	1380 [640–2360]	920 [260–1840]	1240 [550–2130]	1510 [800–2480]	1710 [850–2640]	< 0.001
Heart rate (^−1^/min)	119 [100–135]	116 [100–134]	113 [96–130]	119 [101–134]	124 [110–140]	< 0.001
SOFA	9 [6–11]	10 [7–12]	8 [6–11]	8 [6–11]	9 [6–11]	< 0.001
Septic shock (Sepsis-3 criteria)	3513 (53,7)	973 (66,7)	938 (48,7)	967 (50,9)	635 (50,5)	< 0.001
Quality of care						
Blood cultures drawn	5342 (81.7)	840 (80.6)	1266 (74.8)	2061 (80.8)	1175 (93.4)	< 0.001
Time to antibiotics (min)	98 [15–305]	115 [20–305]	103 [10–350]	105 [15–320]	80 [15–240]	0.02
Outcome						
New onset dialysis	1502 (23.0)	368 (35.3)	374 (22.1)	538 (19.7)	222 (20.7)	< 0.001
ICU mortality	1952 (29.9)	444 (42.7)	528 (31.3)	677 (26.6)	303 (24.1)	< 0.001
28-Day mortality	2024 (31.8)	467 (45.7)	539 (32.6)	693 (28.0)	325 (26.6)	< 0.001
Hospital mortality	2448 (37.5)	546 (52.5)	676 (40.0)	850 (33.4)	376 (30.0)	< 0.001

Data are expressed as median [Q1–Q3] or number and percentage, *n* (%); *p* values for comparison between body temperature groups by Chi-square or Kruskal–Wallis test

*BC* blood culture, *PCT* procalcitonin, *CRP* C-reactive protein, *WBC* white blood cell count, *SOFA* Sequential Organ Failure Assessment Score

### Average outside temperature

**Fig. 3:**
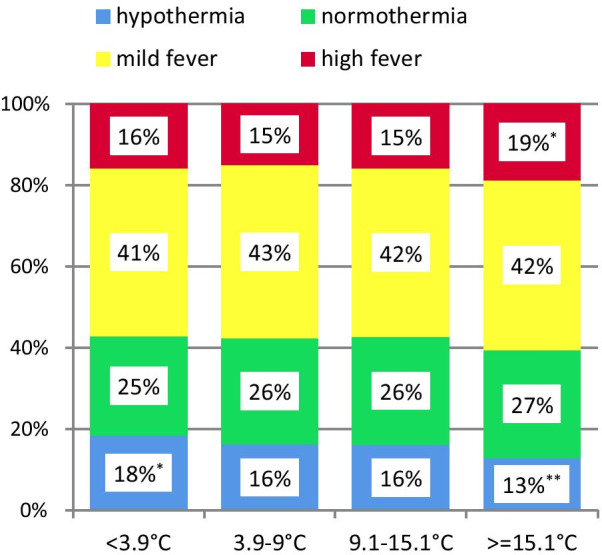
Frequency of body temperature groups depending on mean outside temperature quartiles up to two days before sepsis onset. *p* = 0.002 for overall difference, superscript *denote cells significantly different from the expected frequencies (*p < 0.01, **p < 0.001) assessed by adjusted standardized residuals

Mean local outside temperature before and at sepsis onset was significantly associated with body temperature (*p* < 0.001, see Additional file 2: aFigure 3&4). With low outside temperatures there was more hypothermia; with high outside temperature there were less hypothermia and more high fever (Fig. 3). Mean outside temperature was not associated with mortality (see Additional file 2: aTable2).

### Predictors of hypothermia, normothermia and fever

A multinomial logistic regression model identified predictors independently associated with the described temperature groups (Table 2). Community-acquired sepsis, an abdominal focus of infection, a lower BMI, higher age and lower outside temperatures were independently associated with hypothermia. A pathogen in blood culture, community or ICU-acquired sepsis and high procalcitonin values were associated with high fever.

**Table 2:** Predictors of body temperature groups—multinominal logistic regression

Variable	Hypothermia ≤ 36.5 °C	Normothermia 35.6–37.5 °C	Mild fever 37.6–39 °C	High fever ≥ 39.1 °C
Pathogen in BC	0.96 (0.80–1.17)	0.79 (0.67–0.93)^**^	Reference category	1.74 (1.46–2.08)^***^
Origin of infection				
Community	1.37 (1.13–1.66)^**^	1.09 (0.92–1.28)		1.34 (1.09–1.65)^**^
ICU	0.50 (0.39–0.64)^***^	0.66 (0.54–0.80)^***^		1.44 (1.15–1.82)^**^
Ward/nursing home	Reference	Reference		Reference
Focus of infection				
Respiratory	0.72 (0.56–0.92)^*^	0.77 (0.62–0.95)^*^		0.89 (0.71–1.11)
Abdominal	1.32 (1.04–1.68)^*^	1.38 (1.12–1.69)^**^		0.44 (0.34–0.56)^***^
Urogenital	0.86 (0.59–1.27)	1.20 (0.88–1.63)		0.95 (0.69–1.31)
Bones/soft tissue	1.38 (0.99–1.91)	1.39 (1.05–1.85)^*^		0.76 (0.54–1.08)
Other/unknown	Reference	Reference		Reference
Age (per 10 years)	1.11 (1.04–1.18)^***^	1.15 (1.09–1.22)^***^		0.87 (0.82–0.92)^***^
BMI (per 5 points)	0.82 (0.76–0.87)^***^	0.92 (0.88–0.97)^**^		1.03 (0.99–1.08)
Procalcitonin log10	0.96 (0.85–1.08)	1.01 (0.92–1.12)		1.43 (1.28–1.60)^***^
Mean temperature (per 10 °C)	0.83 (0.74–0.93)^**^	1.01 (0.91–1.11)		1.12 (0.99–1.25)

Multinomial hierarchical logistic regression model with random intercepts for study centers for the prediction of body temperature category based on 5166 cases with all necessary data available

Leucopenia, leucocyte count and sex were not significantly associated and omitted from the final model. *p* values were < 0.001 for all predictors included in the model. ^*^Denotes predictors significantly associated (^*^p < 0.05, ^**^p < 0.01, ^***^p < 0.001) with the body temperature category

### Mortality

**Fig. 4:**
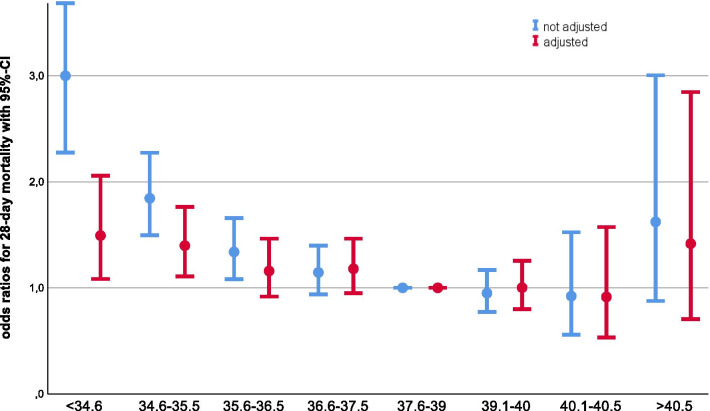
Odds ratios for 28-day mortality from a logistic regression model without and with adjustment for other predictors of mortality (see Additional file 2: aT1&aT2)

28-Day mortality was highest (45.7%) in hypothermic patients and lowest (27.1%) in patients with high fever (*p* < 0.001) (Table 1, see Additional file 2: aFigure 5). Looking at smaller body temperature intervals, a decreased mortality could be seen with higher body temperature; for fever > 40.5 °C there might be a trend toward an increased mortality but without any statistical significance (Fig. 4, see Additional file 2: aTable 2, aFigures 6&7). After adjustment for baseline variables and disease severity, the increased mortality risk associated with hypothermia was less pronounced while there was no relevant change in the mortality risk for patients with fever (Fig. 4, see Additional file 2: aTables 1&2). Repeating the analysis with the four body temperature categories showed similar results (Additional file 2: aTables 3&4).

## Discussion

Using a large dataset, we observed a bimodal distribution of body temperature in septic patients. We could confirm an increased mortality associated with hypothermia that was less pronounced after adjustment for disease severity. Community-acquired sepsis, age, lower BMI and an abdominal focus of infection were associated with hypothermia, while bacteremia and a high procalcitonin level were associated with higher fever. Environmental temperature has an influence on body temperature reaction in human sepsis patients.

The definition of normothermia seems to be mostly dependent on age and site of measurement [20]. Our interval of 35.6–37.5 °C was chosen based on the elderly patients in our dataset, clinical judgment and the advantage of using 0.5 °C intervals for analysis and readability. The second frequency peak around 35.5 °C observed in our data is around the lower limit of normal, but those values would be rare in a healthy population. The mean body temperature in such a population would be slightly above 36.5 °C [20], a value around an observed frequency nadir in our dataset. The obvious interpretation is that severely septic patients show either a hypothermic or more frequently a fever response. The presentation of a portion of septic patients with hypothermia is common knowledge and has been part of the SIRS definition [6], but to our knowledge the bimodal pattern of hyper- or hypothermic response where normothermic patients are a rarity has not been described that clearly before.

Fever is a physiological and strongly preserved reaction to infection slowing the growth of bacteria, stopping the growth of most fungi and enhancing bacterial killing by immune cells [2, 22, 23]. Pathogens in blood culture and higher procalcitonin levels, both associated with high fever in our data, might represent a degree of pathogen challenge where a strong immune resistance is most beneficial. Previous studies have shown conflicting results regarding an association of circulating TNF-α or several interleukins with the type of thermoregulatory response in septic patients [24–27]. But procalcitonin kinetics might be better suited to measure inflammatory activation than TNF-α or interleukins whose peak values are before the first blood draw in most clinical scenarios [28]. The mechanisms regulating hypothermia, which involve much of the same cytokines that regulate fever, are not well understood [10, 24–27]. In animal experiments, a thermoregulatory reaction to sepsis dependent on environmental temperature has been observed [9, 10]. This has been interpreted as poikilothermia, meaning a dissociation of the upper and lower threshold of temperature regulation where body temperature becomes dependent on outside conditions [9]. But recent data indicate a well-regulated mechanism where activated immunity is in an energetic trade-off with homeothermy [10]. Under cold conditions or calorie restriction, mice show a hypometabolic response promoting tolerance, while warm and well-fed mice show a normothermic response promoting resistance but associated with a higher mortality [10]. In our data, a lower BMI and older age, both surrogates of lower metabolic fitness, were associated with hypothermia in line with previous findings from a smaller cohort [25]. Further factors associated with hypothermia (abdominal focus) or its absence (ICU-acquired sepsis) are difficult to interpret as abdominal surgery might promote hypothermia and ICU care is associated with multiple manipulations of active warming, administration of energy substrates and interventions aimed at the restoration of cardiopulmonary homeostasis already in the early phase of disease.

To our knowledge, we are the first to show an influence of environmental temperature on the thermoregulatory response in septic humans while a hypothermic response to a cold environment depending on metabolic reserves has been described long ago [29], and an adaptive hypometabolic state in critical illness has been theoretically discussed recently [30]. The average outside temperature for the hospital location is only a proxy for the situation of the individual patient, which is dependent on local weather at the place and time of disease onset, housing, heating and clothing. In hospital-acquired sepsis, the exposure to cold conditions should be limited, and community-acquired sepsis was indeed associated with hypothermia. When it comes to warmer outside temperatures, one should keep in mind that private homes, normal hospital wards and even some ICUs in Germany are not regularly air-conditioned, resulting in a direct association of warm outside and indoor temperatures. Being able to show an effect with such an imperfect proxy might indicate that the real effect must be quite large as it is still discernible from the statistical noise.

Opposed to mice, hypothermia in human sepsis is associated with higher mortality in our data and the literature [11]. But compared to animal experiments, where only caloric intake or environmental temperature is manipulated, human patients are much more heterogeneous. Age, for example, is associated with hypothermia and worse outcome. After adjusting for factors associated with the type of thermoregulatory response and severity of illness, the association between hypothermia and mortality was much less pronounced but still present. Therefore, the hotly debated question whether and in which patients’ high fever or hypothermia is an adaptive or a maladaptive and possibly detrimental response [9, 31, 32] remains open. But the question is of utmost importance as there is a tendency to correct body temperature toward a perceived normal by physical or pharmacological means as part of routine care and in clinical trials [33–37]. For the treatment of fever, there is relatively good data that in general, patients do not benefit from temperature control [35, 37] even though it is widely used [38], but patients with a fever above 40 °C are barely represented in those studies. While inducing hypothermia is probably harmful [36], there are no clinical data proving that hypothermic patients profit from active warming measures aiming for normothermia or mild fever. Only a small pilot study is dealing with the question so far (NCT02706275).

Some differences in quality of care depending on body temperature are in line with previous findings [39, 40]. Not surprisingly in the presence of fever, physicians are quicker to administer antimicrobials and to draw blood cultures, especially as they expect a higher rate of pathogen detection than in febrile patients [41, 42].

Our analysis has several strengths and weaknesses. To our knowledge, it is the first study on the subject using a large number of cases to look at several degrees of fever and hypothermia and to look at environmental temperature as an associated factor. As this is a secondary analysis of data from a multicenter quality improvement trial, the available information is limited. Unfortunately, we have no detailed information on oxygen delivery or consumption, no detailed medical history with potentially relevant comorbidities, no information on antipyretic medication or sedatives, no cytokine panel, no information on the patients’ real environmental conditions and no serial temperature measurements. Therefore, we cannot use temperature trajectories to further distinguish our patient groups [43]. Temperature was also measured in an unstandardized way in different body locations. Even though to our knowledge there has been no systematic change in temperature management over the last ten years, our dataset might not be fully representative of today’s patients due to changes in sepsis management. Confounding due to differences on the hospital level is of special concern when analyzing outside temperature, which is clearly associated with a hospital’s location. We took clustering into account by including random intercepts into our regression analyses but could not assess the influence of specific hospital factors with forty clusters.

## Conclusion

Fever and hypothermia are the two different responses in human sepsis while normothermia is rare. Even though associated with several factors including environmental conditions, their regulatory causes and their potential impact on outcome is poorly understood, more detailed observational studies are needed to understand those before the therapeutic interventions targeting body temperature or metabolic adaptation in general can be tested in the right target population.

## Supplementary Information

**Additional file 1:** All involved ethical bodies with reference number of the vote.

**Additional file 2:** Additional tables and figures.

## Data Availability

The datasets analyzed during the current study are available from the corresponding author on reasonable request.

## Abbreviations

PCT: Procalcitonin
ICU: Intensive care unit
CI: Confidence interval
IQR: Interquartile range
CRP: C-reactive protein
WBC: White blood cell count
SOFA: Sequential Organ Failure Assessment Score
ANOVA: Analysis of variance
BMI: Body mass index
