# Supplementary material for: Fever and hypothermia represent two populations of sepsis patients and are associated with outside temperature
Source: Crit Care. 2021 Oct 21;25:368. doi: 10.1186/s13054-021-03776-2 (PMC8532310; doi:10.1186/s13054-021-03776-2)
Supplement: Supplementary file 2 — Additional file 2: Additional tables and figures. [file 13054_2021_3776_MOESM2_ESM.docx]

Additional file 2: Additional tables and figures

# Additional Tables

aTable 1 Association of body temperature intervals with 28-day mortality

| **Variable** | **Odds ratio** | **95% CI** | **p-Value** |
| --- | --- | --- | --- |
| <34.6 | 3.00 | 2.27-3.95 | <.001 |
| 34.6-35.5 | 1.84 | 1.49-2.27 | <.001 |
| 35.6-36.5 | 1.34 | 1.08-1.65 | 0.01 |
| 36.6-37.5 | 1.14 | 0.93-1.39 | 0.2 |
| 37.6-39 | reference |  |  |
| 39.1-40 | 0.95 | 0.77-1.16 | 0.6 |
| 40.1-40.5 | 0.92 | 0.55-1.52 | 0.8 |
| >40.5 | 1.62 | 0.87-3.00 | 0.1 |

Results of a logistic hierarchical linear model adjusting for clustering of cases in hospitals for the influence of body temperature intervals (°C) on 28-day mortality; based on the 4552 cases with all available data as in aTable2; test of overall effect of body temperature was significant (p<0.001); Nagelkerkes Rsquare was 0.03.

aTable 2 Binary logistic regression model – association of body temperature intervals and other factors with 28-day mortality

| **Variable** | **Odds ratio** | **95% CI** | **p-Value** |
| --- | --- | --- | --- |
| <34.6 | 1.49 | 1.08-2.05 | 0.01 |
| 34.6-35.5 | 1.40 | 1.10-1.76 | 0.005 |
| 35.6-36.5 | 1.16 | 0.91-1.46 | 0.2 |
| 36.6-37.5 | 1.18 | 0.95-1.46 | 0.1 |
| 37.6-39 | reference |  |  |
| 39.1-40 | 1.00 | 0.80-1.25 | 1.0 |
| 40.1-40.5 | 0.91 | 0.53-1.57 | 0.7 |
| >40.5 | 1.42 | 0.70-2.84 | 0.3 |
| Age (per 10 years) | 1.34 | 1.27-1.42 | <.001 |
| logPCT | 0.81 | 0.72-0.89 | <.001 |
| BMI (per 5 points) | 0.90 | 0.85-0.95 | <.001 |
| Ward acquired | reference |  |  |
| Community-acquired | 0.88 | 0.74-1.03 | 0.1 |
| ICU- acquired | 1.19 | 0.98-1.43 | 0.1 |
| Pathogen in BC | 1.21 | 1.03-1.42 | 0.01 |
| Lactate (per 1mmol/l) | 1.12 | 1.09-1.14 | <.001 |
| SOFA score (per point) | 1.16 | 1.13-1.19 | <.001 |
| Other/unknown foci of infection | reference |  |  |
| Respiratory focus of infection | 0.96 | 0.78-1.16 | 0.7 |
| Abdominal focus of infection | 0.76 | 0.62-0.93 | 0.001 |
| Urogenital focus of infection | 0.47 | 0.34-0.65 | <.001 |
| Bones/soft tissue focus of infection | 0.96 | 0.72-1.27 | 0.8 |
| Sex (male) | 0.88 | 0.76-1.01 | 0.1 |
| Septic shock | 1.07 | 0.89-1.27 | 0.8 |
| Leucopenia (<4Gpt/l) | 1.35 | 1.05-1.72 | 0.02 |
| Leucocyte count (per Gpt/l) | 1.01 | 0.99-1.01 | 0.1 |
| Mean outside temperature (per 1°C) | 1.00 | 0.99-1.01 | 0.6 |
| Time to antibiotics (per hour) | 1.00 | 0.99-1.00 | 0.4 |

Results of a logistic hierarchical linear model adjusting for clustering of cases in hospitals for the influence of body temperature intervals (°C) and potential confounders on 28-day mortality; based on 4552 cases with all available data; test of overall effect of body temperature was not significant (p = 0.054); Nagelkerkes Rsquare was 0.23.

aTable 3 Binary logistic regression model – association of body temperature categories with 28-day mortality

| **Variable** | **Odds ratio** | **95% CI** | **p-Value** |
| --- | --- | --- | --- |
| hypothermia | 2.17 | 1.81-2.59 | <.001 |
| normothermia | 1.23 | 1.04-1.44 | 0.01 |
| mild fever | reference |  |  |
| high fever | 0.98 | 0.8-1.18 | 0.8 |

Results of a logistic hierarchical linear model adjusting for clustering of cases in hospitals for the influence of body temperature categories on 28-day mortality; based on 4552 cases with all available data as in aTable4; test of overall effect of body temperature was significant (p<0.001); Nagelkerkes Rsquare was 0.02.

aTable 4 Binary logistic regression model – association of body temperature category and other factors with 28-day mortality

| **Variable** | **Odds ratio** | **95% CI** | **p-Value** |
| --- | --- | --- | --- |
| hypothermia | 1.43 | 1.16-1.75 | <.001 |
| normothermia | 1.17 | 0.98-1.39 | 0.08 |
| mild fever | reference |  |  |
| high fever | 1.01 | 0.82-1.25 | 0.9 |
| Age (per 10 years) | 1.34 | 1.26-1.41 | <.001 |
| logPCT | 0.81 | 0.72-0.89 | <.001 |
| BMI (per 5 points) | 0.90 | 0.85-0.95 | <.001 |
| Ward aquired | reference |  | 0.001 |
| Community-acquired | 0.88 | 0.74-1.03 | 0.1 |
| ICU- acquired | 1.19 | 0.98-1.43 | 0.1 |
| Pathogen in BC | 1.22 | 1.04-1.42 | 0.01 |
| Lactate (per 1mmol/l) | 1.12 | 1.09-1.14 | <.001 |
| SOFA score (per point) | 1.16 | 1.13-1.19 | <.001 |
| Other/unknown foci of infection | reference |  |  |
| Respiratory focus of infection | 0.96 | 0.79-1.16 | 0.7 |
| Abdominal focus of infection | 0.76 | 0.62-0.93 | 0.001 |
| Urogenital focus of infection | 0.47 | 0.34-0.65 | <.001 |
| Bones/soft tissue focus of infection | 0.96 | 0.72-1.27 | 0.8 |
| Sex (male) | 0.88 | 0.76-1.01 | 0.1 |
| Septic shock | 1.07 | 0.89-1.27 | 0.5 |
| Leucopenia (<4GPt/l) | 1.35 | 1.05-1.72 | 0.02 |
| Leucocyte count (per Gpt/l) | 1.01 | 0.99-1.01 | 0.1 |
| Mean outside temperature (per 1°C) | 1.00 | 0.99-1.01 | 0.6 |
| Time to antibiotics (per hour) | 1.00 | 0.99-1.00 | 0.4 |

Results of a logistic hierarchical linear model adjusting for clustering of cases in hospitals for the influence of body temperature categories and potential confounders on 28-day mortality; based on 4552 cases with all available data; test of overall effect of body temperature was significant (p = 0.005); Nagelkerkes Rsquare was 0.21.

Additional file 2 – Additional Figures


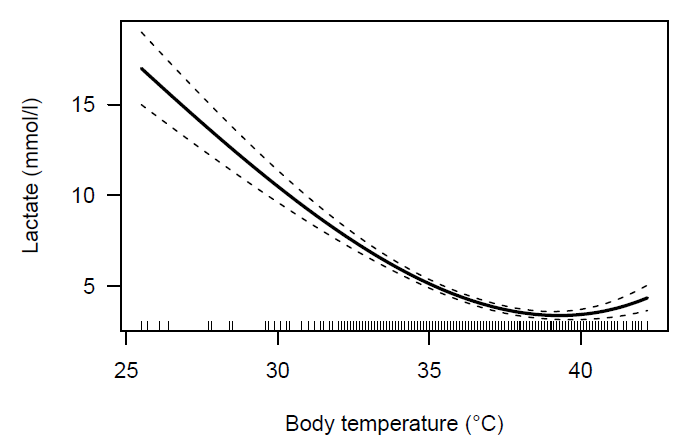


p<0.001

**aFigure 1: Graphical depiction of fractional polynomial analysis for the association of body temperature with lactate; p-value for the overall significance of the association.** **Dashed lines denote 95%CI’s. One outlier in body temperature with a temperature of < 25°C was excluded.**

p<0.001

**aFigure 2: Graphical depiction of fractional polynomial analysis for the association of body temperature with procalcitonin; p-value for the overall significance of the association.** **Dashed lines denote 95%CI’s. One outlier in body temperature with a temperature of < 25°C was excluded.**

p<0.001

**aFigure 3** **: Graphical depiction of fractional polynomial analysis for the association of body temperature with outside temperature; p-value for the overall significance of the association.** **Dashed lines denote 95%CI’s. One outlier in body temperature with a temperature of < 25°C and four outliers in outside temperature with a temperature of < -15°C were excluded.**

**aFigure 4: Violin Plot for average outside temperature associated with body temperature intervals. p-value for overall difference, each superscript letter denotes a homogenous subgroup with increasing values from a to d, categories not sharing a common letter are significantly different from each other. Kruskal-Wallis-Test with stepwise post-hoc comparison.**

**aFigure 5: Survival curves up to day 28 for body temperature categories. p<0.001 for overall difference.**

**aFigure 6: 28-day mortality rate associated with body temperature intervals (°C). p<0.001 for overall difference, each superscript letter denotes a homogenous subgroup with increasing mortality from a to d, categories not sharing a common letter are significantly different from each other. Chi-Square with z-test post-hoc analysis.**

p<0.001

**aFigure 7** **: Graphical depiction of fractional polynomial analysis for the association of body temperature with 28-day mortality; p-value for the overall significance of the association.** **Dashed lines denote 95%CI’s. One outlier in body temperature with a temperature of < 25°C was excluded.**
